# Comment on “Barrier identification and strategic design for hypertriglyceridemia management in the post-acute coronary syndrome patient population at a large integrated health system”

**DOI:** 10.1016/j.ahjo.2026.100920

**Published:** 2026-09-29

**Authors:** Srikanth Inturi, Anjana Karunakaran Nair, Pramila Chaubey, Grandhi Surendra

**Affiliations:** a Department of Pharmacy, ESIC Hospital, Kakinada, Andhra Pradesh, India; b Department of Pharmaceutics, College of Pharmacy, Shaqra University, Dawadmi, 11961, Saudi Arabia; c School of Pharmacy, ITM University, Gwalior, Madhya Pradesh, India

**Keywords:** Hypertriglyceridemia, Acute coronary syndrome, Icosapent ethyl, Cardiovascular risk reduction, Treatment barriers, Guideline-directed therapy, Clinical decision-making, Integrated health system, Medication management, Quality improvement

Dear Editor,

We read with great interest the study by Salvati et al. [1], on strategies to improve hypertriglyceridemia (HTG) management after acute coronary syndrome (ACS).

## 1 Undefined denominator for the treatment gap

The authors report that 1.1% of 175 patients received icosapent ethyl (IPE) and conclude that use remained minimal. This denominator holds every post-ACS patient with paired triglyceride values, not patients eligible for IPE. Eligibility requires persistent triglyceride elevation on statin therapy with controlled low-density lipoprotein cholesterol, and the report never states how many patients met this criterion [2]. Without this count, 1.1% cannot distinguish a treatment gap from appropriate restraint. Moreover, both IPE recipients had triglycerides above 500 mg/dL, outside the range enrolled in the outcomes trial that established IPE's benefit [3]. The finding therefore describes selective use, not absent use. The clinical implication is direct: a health system may launch a campaign against a gap it has not measured. The statement that 99.4% received medication never names the agent.

## 2 Intervention mismatched to the named barrier

Interviews and the deliberative session identified coverage, prior authorization, and alert fatigue as leading barriers [4]. All six strategies delivered educational or reference material, and none changed coverage; only a note template touched the workflow. The package therefore tested whether lectures could overcome a cost barrier, and a null result was predictable. Four champions, three of whom were pharmacists and none were cardiologists, co-designed it, although one participant warned that messages from pharmacy would go unheard. The interview stage also included only one cardiologist; therefore, prescribers themselves shaped little of the design.

## 3 Conclusions the data do not measure

Seventeen clinic observations recorded no post-ACS patient; therefore, the process maps rest on seven interviews. The pre-implementation survey drew 16 respondents without a stated response rate. The abstract states that the presentation failed to translate into clinician motivation. No instrument measured motivation, and no clinician completed the post-implementation survey. Penetration relied on resource downloads, yet the presentation was recorded and slides were shared; therefore, viewing went untracked. The pilot produced no prescribing change to interpret, whatever the cause. The electronic health record review compared encounters without a comparison clinic or a pre-pilot baseline. Similarly, the abstract calls standardized management acceptable, while acceptability scored lowest (mean 3.5, median 3.8, minimum 1.5), and the text reports its standard deviation as 1.74 against 0.74 in the summary.

## 4 Funding and conclusion

Amarin, the manufacturer of IPE, funded the study, two employees co-authored it and assisted with interpretation, and the package promotes IPE. Independent analysis of prescribing data would remove any dependence on the funder's input. Taken together, the study leaves open whether education alone can change prescribing. It earns its place by mapping local prescribing barriers. An eligibility-defined denominator, a strategy that targets coverage, and a concurrent comparison clinic would allow other health systems to judge the approach before adopting it at scale.

## CRediT authorship contribution statement

**Srikanth Inturi:** Writing – review & editing. **Anjana Karunakaran Nair:** Visualization. **Pramila Chaubey:** Supervision. **Grandhi Surendra:** Conceptualization.

## Informed consent

Not applicable.

## Ethical approval

Not applicable (Commentary on a previously published article).

## Declaration of Generative AI and AI-assisted technologies in the writing process

Generative AI tools were used only for language refinement and formatting assistance. All scientific interpretation, critical appraisal, and manuscript preparation were performed independently by the authors.

## Funding

None.

## Declaration of competing interest

The authors declare that there are no conflicts of interest.

## Data Availability

No new data were generated or analyzed in this study.
